# Is moss-associated nitrogen fixation controlled by the same factors across shoots, species and sites?

**DOI:** 10.1016/j.envexpbot.2025.106262

**Published:** 2025-11

**Authors:** Jørgen Ulrik Graudal Levinsen, Mingyue Yuan, Anders Michelsen, Kathrin Rousk

**Affiliations:** a Department of Biology, University of Copenhagen, Universitetsparken 15, Copenhagen DK-2100, Denmark; b Department of Ecoscience, Marine Ecology Section, Aarhus University, C.F. Møllers Alle 1131, Aarhus DK-8000, Denmark; c Department of Biology, Center for Volatile Interactions (VOLT), University of Copenhagen, Universitetsparken 15, Copenhagen DK-2100, Denmark

**Keywords:** Arctic, Bryophytes, *Hylocomium splendens*, *Pleurozium schreberi*, Nitrogen fixation, Precipitation, Limiting nutrients

## Abstract

Biological N_2_ fixation performed by moss-associated cyanobacteria is among the main sources of new nitrogen (N) input in pristine subarctic ecosystems. Yet, until now there has been a lack of knowledge on the drivers of biological N_2_ fixation on small spatial scales (moss segment) in relation to the drivers at larger scales (moisture ecosystem). To investigate this, we assessed the capacity of N_2_ fixation along shoots of two common moss species, *Pleurozium schreberi* and *Hylocomium splendens,* collected along a steep precipitation gradient in the Subarctic. Furthermore, concentrations of nutrients and pH were analysed along the same moss shoot-gradients. We found the highest nitrogenase activity in *H. splendens* collected at the location with the highest mean annual precipitation. Nitrogenase activity varied significantly along the moss shoots, and between species and location along the precipitation gradient. *P. schreberi* had the highest nitrogenase activity in the middle segments, while for *H. splendens*, it was highest in the lowest segments (below 3 cm). Contents of iron, molybdenum and N generally increased with moss segment depth, but phosphorus concentrations decreased and pH was stable across segments. Taken together, the factors that drive nitrogenase activity at small scales differ between moss species, whereas precipitation predominantly controls nitrogenase activity at larger scales (across habitats).

## Highlights

• Nitrogenase activity varied significantly along moss shoots.
• Different nutrients influenced nitrogenase activity at small scales between moss species.
• Precipitation predominantly controls nitrogenase activity at large scales (across ecosystems).

## 1 Introduction

Biological N_2_ fixation is one of the main pathways for new N to enter pristine terrestrial ecosystems (Chapin et al., 2011). Mosses are widespread in the subarctic and other high latitude, nutrient limited ecosystems, where they can form dense carpets with groundcover up to more than 70 % (DeLuca et al., 2002, Gundale et al., 2012). Feather mosses like *Pleurozium schreberi* and *Hylocomium splendens*, both common in these environments, are colonized by cyanobacteria capable of performing N_2_ fixation (Rousk et al., 2013). Together, they can account for 1–3 kg N fixed ha^−1^ year^−1^ (Rousk and Michelsen, 2017), which equals about 50 % of total N input in these ecosystems (Goth et al., 2019). Yet, N_2_ fixation by moss-associated cyanobacteria is greatly affected by abiotic factors. For instance, moisture, temperature and light can promote moss-associated N_2_ fixation rates (Gundale et al., 2012, Rousk et al., 2017a) but increased N input and low moss pH commonly inhibit it (Gundale et al., 2011, Alvarenga and Rousk, 2021, Wang et al., 2022). Results on the effects of nutrient availability other than N are ambiguous. Molybdenum (Mo) and phosphorus (P) availability have been shown to promote, inhibit or have no effect on moss-associated N fixation and the effects seem to be strongly dependent on time and nutrient addition rates (Zheng et al., 2019, Rousk et al., 2017b, Rousk and Rousk, 2020, Clasen et al., 2025). Due to its importance for the nitrogenase enzyme, iron (Fe) could be another factor driving N_2_ fixation. Indeed, in tropical forests, Fe has been shown to promote nitrogenase activity in the decomposing leaf layer (Winbourne et al., 2017). Hence, N_2_ fixation is significantly affected by biogeochemical factors and climate change, and additions to our understanding of the N-cycle in high latitude ecosystems is key in order to improve predictions for how ecosystem functions will respond to future changes in precipitation, temperature and nutrient availability.

The factors listed above have previously been assessed at the level of entire moss carpets or moss-shoots, but few studies report systematically on the effects at a smaller scale, at different depths, and with that, age of moss shoots, although moisture, temperature and nutrient content are likely to differ along moss shoots. For instance, BNF has been found to be higher in the green, upper 4 cm (younger) than in the yellow-brown (older) parts of moss shoots from *H. splendens* and *P. schreberi* likely due to effects of light (Leppänen et at. 2012). Similarly, the highest numbers of moss-associated, N_2_-fixing cyanobacteria (Renaudin et al., 2022) and BNF activity (Smith, 1984) were found in the top of the moss shoot (1–3 cm from apex). Yet, another study (Gavazov et al., 2010) cut the top 3 cm of *H. splendens* into 1 cm segments and found a pattern of highest BNF in the middle segment, and lower BNF in the top and bottom segments. Taken together, the highest nitrogenase activity seems to be found in the top 3 cm of moss shoots, which likely reflects an environment conducive to cyanobacterial N_2_ fixation. This colonization pattern may correlate with higher nutrient (such as Mo, P and Fe) concentrations in the top of the moss shoot that promote N_2_ fixation. Alternatively, the requirement of BNF for Mo, P and Fe could be higher at the top part, and hence, we would expect lower concentration of those nutrients where we find the highest BNF. To date, we lack data on nutrient distribution along moss shoots, as well as knowledge if this pattern holds true across species and even larger scale such as locations. This can have large implications for ecosystem nutrient budgets as averaged N fixation rates across moss shoots can lead to underestimates of N fixation rates.

Besides nutrients as driving factors for the top-shoot colonization of mosses by cyanobacteria, moisture is likely one of the primary contributing factors at large scale. Dry mosses are dormant and support no BNF activity as the colonizing cyanobacteria are only active when moist, and highest BNF rates are found in sufficiently moist mosses (Rousk et al., 2014). Mosses cannot control water loss, as they have no vascular tissue, making them entirely dependent on moisture from the environment. The effects of moisture might also affect the BNF activity along moss shoots. The upper parts of moss carpets tend to dry out first, thus lowering evaporative water losses and preserving moisture in the lower parts which could maintain BNF activity longer. Hence, BNF may differ significantly within a few cm along a moss shoot, and assessing BNF and nutrient concentrations at shoot level or in entire moss carpets only may not correctly reflect BNF. This can lead to incorrect estimates of N_2_ fixation when upscaling rates to ecosystem level, if BNF is driven by different factors along a moss shoot, which is not accounted for in current predictive models.

The aims of this study were to assess (1) if the legacy of varying precipitation patterns collected at three sites affect nitrogenase activity along moss shoots of the two common feather mosses, *P. schreberi* and *H. splendens,* and (2) to investigate where along a moss shoot BNF takes place and if this is linked to the pivotal factors (Fe, P, N and Mo contents as well as pH) controlling BNF. We hypothesized that (H1) nitrogenase activity is highest at the site that receives the highest precipitation, (H2) highest nitrogenase activity takes place in the middle segments of the moss shoots (below 1 cm) given that moisture and light is optimal here, and (H3) that Fe, Mo and P is positively linked to nitrogenase activity, while N and low pH is negatively related to nitrogenase activity. Finally, (H4) nitrogenase activity at different depths along the moss shoot will converge over time if exposed to similar conditions. To address these hypotheses, we established a common garden experiment in which we divided moss shoots collected from sites that differ greatly in mean annual precipitation in Northen Sweden into 3 segments and measured nitrogenase activity over time, as well as nutrient content and moss pH.

## 2 Materials and methods

### 2.1 Sampling sites, sample collection and sample handling

Moss samples were collected in October 2016 from three locations along a steep precipitation gradient stretching roughly 40 km in subarctic Northern Sweden, near Abisko. The mean annual air temperature in the Abisko area is 0.2 °C with the warmest and coldest months July and February, average temperatures 11.9 and −10.0 °C, respectively (30-year mean 1986–2015, Abisko Scientific Research Station 2016). Collection sites were chosen by selecting sites with a similar vegetation type, a birch forest with a moss-dominated understory. Moss carpets (18 ×18 cm; 10–15 cm depth, including green and brown, senescent parts) were taken by hand and placed in 8 L plastic boxes. Six replicate samples were taken from each of the three sites with at least 5 m distance between them.

The site with the lowest mean annual precipitation (MAP) is close to Abisko Research Station, at 422 m above sea level (a.s.l.) and 300 mm MAP (68°34’N, 18°82’E). The medium precipitation site, Låktatjåkka is at 487 m a.s.l. and receives almost three times the precipitation of the Abisko site, 850 mm MAP and is ca. 30 km west of Abisko (68°42’N,18°32’E). At this site, unfortunately only samples from *P. schreberi* could be obtained. The site with the highest MAP, Katterjåkk, at 473 m a.s.l. receives 1100 mm MAP and is ca. 36 km west of Abisko (68°42’N, 18°18’E). At all sites the sampling was done far enough from roads and railroads (at least 100 m) as not to be impacted by these (Goth et al., 2019).

After transportation to Copenhagen, the samples were kept in growth chambers with 6 h light (300 nmol m^−2^ s^−1^) at a constant temperature of 0°C, reflecting the conditions at the time of sampling in the field. Samples were watered regularly to avoid desiccation and only with double distilled water (DD-H_2_O) to avoid nutrient contamination and to favour nitrogenase activity. The field collection time was late fall just before onset of winter, outside the vascular plant growing season of the area (Lett and Michelsen, 2014), and storage conditions were chosen to maintain the samples in their seasonal state until the start of the experiment.

During the experiment, temperature, moisture and light were kept constant. This was done in order to attribute differences in nitrogenase activity between moss samples to variance in other factors known to affect nitrogenase activity as measured here, namely Fe, Mo, P and N content and pH. Constant and equal temperature, light and moisture for all segments is not expected under natural field conditions. Instead, the top segment is expected to receive more light than segments further down the moss shoot, but is also expected to be subject to larger fluctuations in temperature and moisture. The moisture and temperature conditions for nitrogenase activity were optimal and equal for all segments, and hence, our measurements represent potential nitrogenase activity along the entire moss shoot.

### 2.2 Nitrogenase activity

**Fig. 1:**
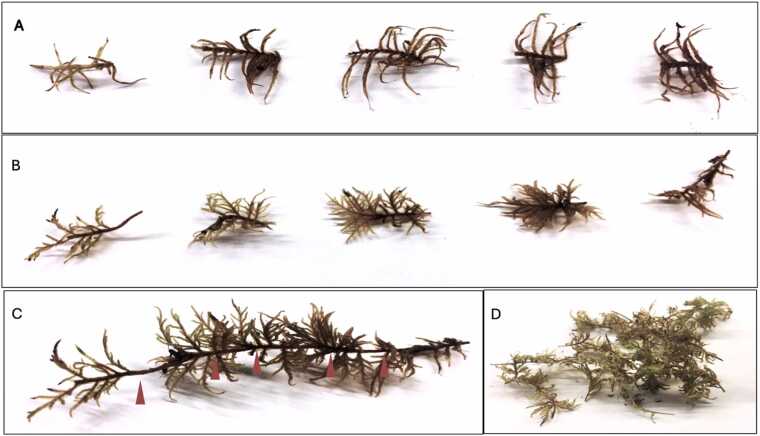
Moss shoots separated according to length. A) *Pleurozium schreberi,* segments from top (left) to bottom (right) and for B) *Hylocomium splendens* where each segment represents one years’ growth from top (left) to bottom (right). C) Intact shoot of *H. splendens* with visible yearly growth segments indicated by red arrows. D) *H. splendens* top segments bundled together to form one sample. Both species were collected at three different locations with varying mean annual precipitation representing dry (Abisko), medium (Låktatjåkka) and wet (Katterjåkk) locations, except Låktatjåkka which only provided samples of *P. schreberi.*

The acetylene reduction assay (ARA) (Hardy et al., 1968) was used to measured nitrogenase activity along the segmented moss shoots from both species from all available locations. The segments were identified first for *H. splendens* as it has a distinct yearly growth (Fig. 1 B, C). *Pleurozium. schreberi* (Fig. 1 A) lacks the same distinct growth pattern and was divided into segments at the same average length, 1 cm, as for *H. splendens*. This choice of segment length for *P. schreberi* corresponds to the annual growth of *P. schreberi* in boreal forests (Lappalainen et al., 2008; Laiho et al., 2011; Rzepczynska et al., 2022), and with the changes in colour seen along the *H. splendens* segments (Fig. 1 A, B). The top green part is the photosynthetic active part, followed by the brown part, where nutrient resorption takes place and the lowest part is the decomposing part of the moss. Both moss species grow in the same habitat and have a similar microclimate, and we assume similar growth rates for both investigated species. Segments (all with an average length of 1 cm) were composited, top segments with top segments, middle segments with middle segments etc. (Fig. 1 D). We kept the field replication (n = 6), with > 30 segments of each of the three segment groups from each species from all sites, Abisko (dry), Låktatjåkka (medium) only for *P. schreberi*, and Katterjåkk (wet), totalling 90 samples (54 for *P. schreberi*, 36 for *H. splendens*). We could obtain enough samples only for the top 3 segments (top 3 cm), as older segments (4–7 cm) were only available for a few shoots and could not provide sufficient material for our experiment.

The moss segments were put into 50 ml transparent plastic tubes and kept in growth chambers set to 18 h light (300 µmol m^−2^ s^−1^) and a constant temperature of 15°C. Regular watering of the moss, using DD-H_2_O, immediately before each incubation was enough to keep the samples moist and to prevent desiccation. The chosen temperature, light and moisture conditions have been shown to be conditions favourable for BNF in feather mosses (Rousk et al., 2017a), also corresponding to the longer daylight period and typical temperature during the growing season in northern Sweden. Our nitrogenase activity results show therefore potential activity under optimal conditions.

For the ARA, airtight rubber septa sealed the tubes and 5 ml of air was replaced with 5 ml acetylene gas (Acetylene gas, technical grade, Air Liquide) using a syringe. This corresponded to 10 % acetylene gas in the tubes. The samples were incubated in the growth chamber for ca. 24 h. After the incubations, 6 ml of air was sampled from each tube and injected into 6 ml pre-vacated vials (Labco, Ceredigion, UK). The rubber septa were removed from the tubes and the tubes were placed in the growth chamber where they were kept until the next incubation. A total of six incubations were completed over 42 days to be able to detect differences in nitrogenase activity over time, with the segments exposed to the same conditions. Incubation frequency was once every week except between incubations four and five with two weeks apart. The analysis of ethylene production was done using a Shimadzu GC-14B Gas Chromatograph equipped with a Porapak N column, a FID detector and with injector, column and detector temperature at 250, 60 and 120°C, using He as carrier gas. To test the natural production of ethylene by mosses, moss samples without acetylene gas were also incubated under the same conditions as described above. No natural production of ethylene was detected in the moss samples, and we subtracted any ethylene residue (mean nmol ethylene (±SE)= 0.08 nmol ±0.001) in the acetylene gas from the ethylene produced by our samples.

After the last ARA incubation, each group of segments was weighed and dried at 60°C. After drying, mosses were weighed again, cut into fine pieces, and analysed for contents of Fe, P, Mo, C and N (below).

### 2.3 Moss nutrient and pH analyses

We analysed moss Fe, P and Mo content using the acid digestion method. Between 90 and 110 mg dry moss was used from each sample, and we used 3 ml of 16 % HNO_3_ diluted to 6 ml 8 % HNO_3_ after digestion and running the Plant Material program on a MARS™ 6 microwave digestor twice to achieve complete digestion. Analyses of Fe, P and Mo contents were done using a Perkin Elmer PinAAcle 900 T Atomic Absorption Spectrometer as in Goth et al. (2019). P and Mo analyses were done using graphite furnace vaporization and Fe was analysed using flame atomic absorption spectrophotometry.

The analysis of total C and N in the moss segments required 4–5.5 mg ground, dry moss packed in tin capsules and was conducted using an Eurovector CN analyser coupled to an Isoprime isotope ratio mass spectrometer, with peach leaves (NIST 1547) as internal standard.

For pH measurements, fresh moss was cut into small pieces and placed in 50 ml transparent plastic tubes with 15 ml DDH_2_O. The tubes were gently shaken, no more than 30 s, to avoid damaging the moss cells and thus measuring pH on a mix of both surface and intracellular pH. Measurements were performed immediately after the shaking. Three replicates (> 20 segments each) were used for the pH measurements for each sample. All samples were measured using a standard pH-meter (PHM240 pH/ION Meter, MeterLab, Radiometer Copenhagen).

### 2.4 Statistical analyses

Differences in nitrogenase activity over time was tested with a liner mixed-effects model with week as fixed effects and sample identity (species, segment and location) as random intercept. The results showed no significant difference in nitrogenase activity across the six weeks (Supporting Table 1). Therefore, the mean of nitrogenase activity across weeks was used in subsequent analyses.

Differences in nitrogenase activity between segments, sites (expressed by their differences in MAP), including interactions between factors, were tested in a model that included nutrient element concentrations (Mo, Fe, P and N) and species as covariates. Variations in contents of Mo, Fe, P and N as well as pH between species and segments and interactions were tested with two-way ANOVA for Abisko (dry) and Katterjåkk (wet), where both moss species could be collected. One-way ANOVA was used to test for differences between segments for Låktatjåkka (medium) which only provided one species*, P. schreberi*. Normal distribution of data and homogenous variance in data were tested with diagnostics plots and log transformation of the acetylene reduction data was performed to achieve normality and homogeneity. All data analyses were done in R (R Core Team. 2020).

## 3 Results

### 3.1 Nitrogenase activity across moss shoots and species

**Fig. 2:**
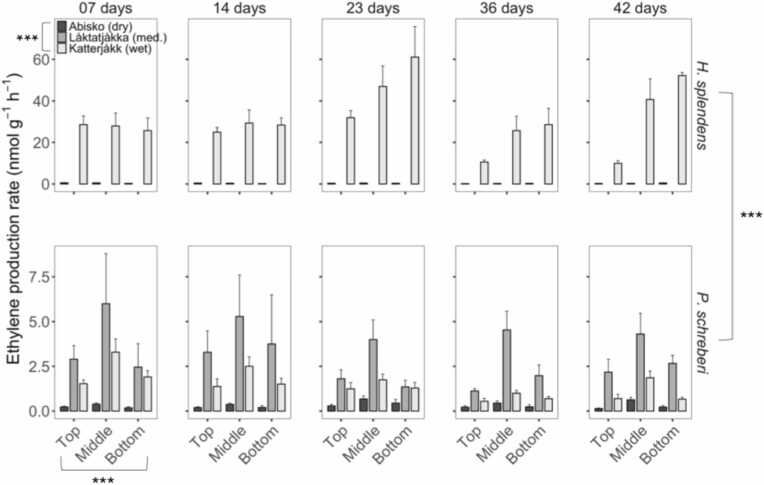
Ethylene production rate ±SE (n = 6) measured over 42 days in three different moss segments from two different species of feather moss. Species and sites are the same as in Fig. 1. Statistical comparisons are provided in Table 1, Table 2. Asterisks (***) indicate p < 0.001.

Nitrogenase activity varied strongly between species, segments and sites, with interactions between species and segments and between species and sites, but not between segments and sites (Fig. 2; Table 1). Nitrogenase activity ranged from 0.11 to 64 nmol g⁻¹ h⁻¹ across all sites, segments and moss species. The highest ethylene production i.e., nitrogenase activity was found at the wettest site (Katterjåkk) after 23 days in *H. splendens* in the lowest segment (the bottom) with a production of 61.1 ± 14.7 nmol g^−1^ h^−1^, and the lowest in the driest site (Abisko) after 42 days in *P. schreberi* in the top segment with a production of 0.14 ± 0.03 nmol g^−1^h^−1^. There was a 10-fold increase in overall ethylene production between the driest site (Abisko) (0.35 ± 0.02 nmol g^−1^h^−1^) and the medium wet site (Låktatjåkka) (3.2 ± 0.4 nmol g^−1^ h^−1^) and another 5-fold increase for both mosses at the wettest site (16.5 ± 1.6 nmol g^−1^h^−1^; Fig. 2). *H. splendens* had larger ethylene production rates than *P. schreberi*, but this was only clear in the samples from the wettest site, where the difference was more than 10-fold between species with an ethylene production of 1.5 ± 0.1 and 31.5 ± 2.1 nmol g^−1^ h^−1^for *P. schreberi* and *H. splendens*, respectively (Fig. 2). There was a consistent pattern of higher ethylene production in the middle segments of *P. schreberi* across all locations and incubation times. For *H. splendens*, ethylene production was highest in the lowest segments, albeit not significantly so (Fig. 2). The patterns in nitrogenase activity remained stable during the experiment with no statistically significant change over time.

**Table 1:** Linear model results showing P-values and F-test values for differences in nitrogenase activity measured by acetylene reduction assay (ARA) between moss species, segments, site-specific mean annual precipitation (MAP) and their interactive effects, and impact of covariates: the tissue contents of iron (Fe), molybdenum (Mo), phosphorus (P) and nitrogen (N) and pH in samples of *H. splendens* and *P. schreberi* collected at field sites with varying mean annual precipitation (MAP): Abisko 300 mm MAP (dry), Låktatjåkka 850 mm MAP (medium) and Katterjåkk 1100 mm MAP (wet), with shoots divided into three segments depths (1–3 cm).

**Parameter**	**Fixed effect**	**df**	**F**	**P**
Nitrogenase activity (measured by ARA)	Species	1	88.9	**< 0.001**
	Segment	2	14.7	**< 0.001**
	Site MAP	2	1107.6	**< 0.001**
	Fe	1	44.2	**< 0.001**
	Mo	1	9.6	**0.002**
	P	1	119.5	**< 0.001**
	N	1	5.4	**0.021**
	pH	1	0.7	0.388
	Species x Segment	2	8.6	**< 0.001**
	Species x Site MAP	1	116.3	**< 0.001**
	Segment x Site MAP	4	1.6	0.228

Bold values indicate statistically significant effects (P < 0.05)

### 3.2 Moss nutrients and pH

**Fig. 3:**
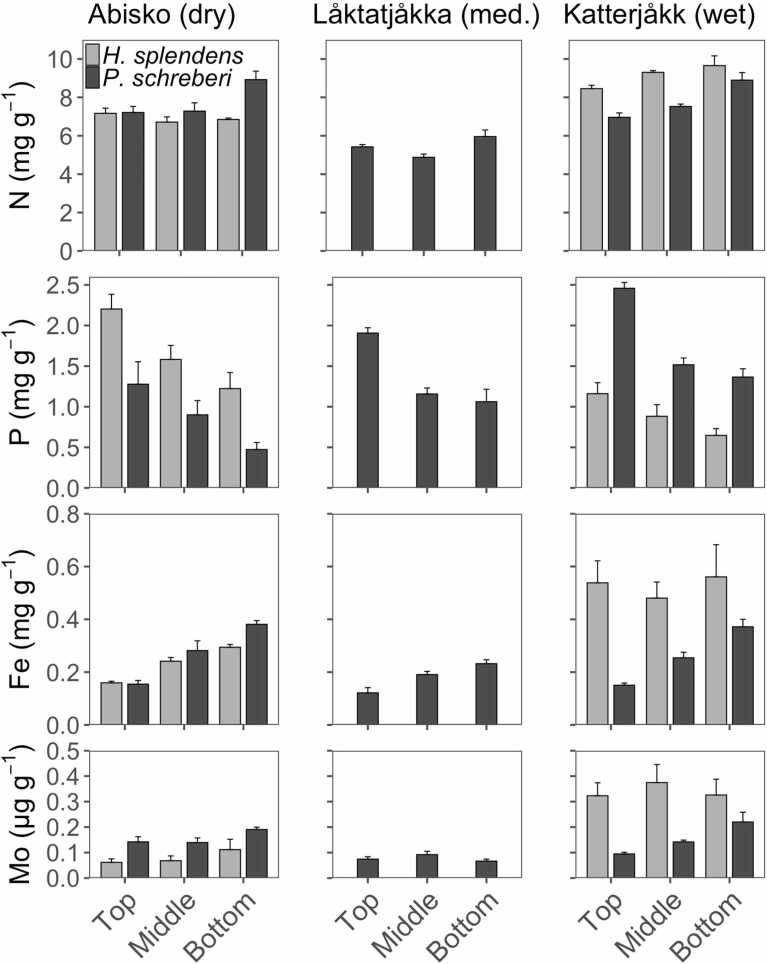
Concentrations of nitrogen, phosphorus, iron and molybdenum ±SE (n = 6) in three different moss segments from two different species of feather moss. Species and sites are the same as in Fig. 1. All units are based on dry weight. Statistical comparisons are provided in Table 1, Table 2.

Contents of Fe and Mo varied along the sites (but less pronounced than the ethylene production), and contents of Fe, P and Mo also varied within sites between species as well as across the moss segments within species (Fig. 3; Table 2). For instance, contents of Fe ranged from 0.56 ± 0.12 mg g^−1^ DW in the bottom segment in *H. splendens* from the wettest site, to a low of 0.12 ± 0.2 mg g^−1^ DW in the top segment in *P. schreberi* from the medium wet site. Overall, the content of Fe varied between sites, species and segments (Fig. 3; Table 2). There was a clear pattern of increasing Fe content with increasing segment depth for *P. schreberi* across all locations, but no clear pattern was found for *H. splendens* (Fig. 3, Table 2).

**Table 2:** Linear model results showing P-values and F-test values for tissue contents of iron (Fe), molybdenum (Mo), phosphorus (P) and nitrogen (N) as well as carbon:nitrogen ratio (C:N) and pH between moss segments, species and their interactive effects for samples of same species and sites as in Table 1.

**Parameter**	Fixed effect	Abisko (dry)			Låktatjåkka (medium)			Katterjåkk (wet)		
		**df**	**F**	**P**	**df**	**F**	**P**	**df**	**F**	**P**
Fe	Segment	2	53.4	**< 0.001**	2	11.2	**0.001**	2	7.2	**0.003**
	Species	1	3.1	0.087	NA	NA	NA	1	53.6	**< 0.001**
	Seg x Spec	2	2.1	0.139	NA	NA	NA	2	6.7	**0.004**
Mo	Segment	2	2.4	0.110	2	1.6	0.238	2	4.4	**0.022**
	Species	1	22.4	**< 0.001**	NA	NA	NA	1	50.4	**< 0.001**
	Seg x Spec	2	0.1	0.897	NA	NA	NA	2	4.0	**0.028**
P	Segment	2	9.3	**< 0.001**	2	12.0	**< 0.001**	2	12.1	**< 0.001**
	Species	1	24.8	**< 0.001**	NA	NA	NA	1	51.8	**< 0.001**
	Seg x Spec	2	0.4	0.666	NA	NA	NA	2	0.3	0.717
N	Segment	2	3.9	**0.030**	2	5.4	**0.018**	2	16.3	**< 0.001**
	Species	1	10.8	**0.003**	NA	NA	NA	1	37.8	**< 0.001**
	Seg x Spec	2	4.8	**0.016**	NA	NA	NA	2	2.5	**0.010**
C:N	Segment	2	3.3	0.052	2	5.6	**0.015**	2	15.8	**< 0.001**
	Species	1	14.1	**< 0.001**	NA	NA	NA	1	24.1	**< 0.001**
	Seg x Spec	2	4.9	**0.014**	NA	NA	NA	2	2.5	0.101
pH	Segment	2	2.1	0.169	2	0.7	0.541	2	0.1	0.929
	Species	1	14.8	**0.002**	NA	NA	NA	1	17.7	**0.001**
	Seg x Spec	2	0.1	0.893	NA	NA	NA	2	1.0	0.383

Bold values indicate statistically significant effects (P < 0.05)

Molybdenum content ranged between 0.37 ± 0.07 µg g^−1^ DW in the middle segments of *H. splendens* from the wettest site and 0.06 ± 0.01 µg g^−1^ DW in the top segment of *H. splendens* from the driest site. Overall, the content of Mo showed a pattern similar to Fe, with strong differences between sites, within sites between species and between segments within species (Fig. 3, Table 3), although with a less pronounced trend towards increasing content with increasing segment depth.

**Table 3:** Moss C:N-ratios (N = 6) and pH (n = 3) content for samples of same species and sites as in Table 1. Given are means ± SE.

**Parameter**	**Species**	**Segment**	Abisko (dry)	Låktatjåkka (medium)	Katterjåkk (wet)
C:N	*H. splendens*	Top	65.74 (2.44)	NA	54.33 (0.80)
		Middle	70.14 (2.99)	NA	50.09 (0.75)
		Bottom	69.04 (0.93)	NA	47.68 (2.53)
	*P. schreberi*	Top	64.45 (2.85)	86.98 (1.77)	67.14 (2.90)
		Middle	64.04 (4.16)	94.60 (3.38)	61.14 (1.59)
		Bottom	51.78 (2.56)	77.43 (5.28)	50.34 (2.65)
pH	*H. splendens*	Top	4.53 (0.08)	NA	5.02 (0.13)
		Middle	4.44 (0.05)	NA	5.29 (0.09)
		Bottom	4.59 (0.02)	NA	5.16 (0.30)
	*P. schreberi*	Top	4.73 (0.10)	4.29 (0.04)	4.73 (0.10)
		Middle	4.66 (0.04)	4.31 (0.06)	4.57 (0.08)
		Bottom	4.76 (0.05)	4.39 (0.08)	4.63 (0.05)

The content of P varied little between sites but greatly between segments and species within sites, with the highest P content at 2.46 ± 0.07 mg g^−1^ DW, found in the top segment of *P. schreberi* from the wettest site and the lowest P content found in the bottom segment of *H. splendens* from the driest site, at 0.47 ± 0.09 mg g^−1^ DW (Fig. 3; Table 3) and there was a pattern of decreasing P with increasing depth for all sites and both species.

Nitrogen content halved from a high of 9.65 ± 0.51 mg g^−1^ DW in the bottom segment of *H. splendens* from the wettest site, to a low of 4.88 ± 0.17 mg g^−1^ DW in the middle segment of *P. schreberi* from the medium wet site. Overall, moss N content was highest in the wettest site and in *H. splendens* from this location but the differences between sites were relatively small (Fig. 3; Table 3). Besides, *H. splendens* from the wettest site had higher concentrations of Fe, Mo and N while *P. schreberi* from this site had the highest content of P.

pH ranged from 4.44 to 5.29, and varied across the precipitation gradient, but generally varied less between segments than between species within sites (Table 3). The pH was lower in *H. splendens* than in *P. schreberi* at the dry site but was higher in *H. splendens* than in *P. schreberi* at the wet site (Table 2).

### 3.3 Correlations between BNF and nutrient content and precipitation along the moss shoots

All nutrient elements (Mo, Fe, N, P) impacted nitrogenase activity, while pH had no significant impact on nitrogenase activity (Table 1). However, in pairwise correlations between nitrogenase activity and the elements, there was generally weak or no correlations across sites and species, except for a few specific instances (Supporting figure 1). For *P. schreberi*, only the content of Mo at the medium wet site had a significant positive effect on nitrogenase activity (R^2^ = 0.40, P = 0.005). For *H. splendens* there were several significant correlations between nitrogenase activity and measured elements. Nitrogen (R^2^ = 0.23, P = 0.04) content correlated positively with nitrogenase activity at the wet site, while P content had a negative correlation at both the dry (R^2^ = 0.23, P = 0.04) and the wet site (R^2^ = 0.32, P = 0.01, Supporting figure 1).

Mean annual precipitation at the three locations Abisko (300 mm), Låktatjåkka (850 mm) and Katterjåkk (1050 mm) could not be correlated with BNF for *H. splendens* as it was only present in two locations. For *P. schreberi* the correlation between MAP and BNF was not linear but instead seemed saturated at the site with the medium MAP (Supporting figure 2).

## 4 Discussion

### 4.1 Nitrogenase activity across sites with different precipitation patterns

We hypothesized (H1) that nitrogenase activity would be higher in mosses collected at the wettest site compared to the driest site. We could only partly confirm this as we found indeed the highest nitrogenase activity at the wettest site for *H. splendens*, where activity was up to 100-fold higher here than in mosses collected at the driest site (Fig. 2). For *P. schreberi*, however, activity was highest at the medium wet site, but still higher at the wettest site than at the driest site. Further, nitrogenase activity was also consistently higher for *H. splendens* compared to *P. schreberi* across all sites. These findings confirm previous results highlighting the importance of moisture for BNF in mosses (Rousk et al., 2014, Rousk et al., 2017a) and moss species-specific differences in associated N_2_ fixation rates (Holland-Moritz et al., 2021). For instance, large variation in nitrogenase activity and associated dominant cyanobacteria has been found for the same moss species investigated here (Warshan et al., 2016) and cyanobacterial communities seem to be host specific (Ininbergs et al., 2011) and may have different requirements for sustaining nitrogenase activity.

Nutrient content (Mo, Fe) was higher in *H. splendens* collected from the wettest site, which could also promote nitrogenase activity as shown elsewhere, also in arctic tundra (Rousk et al., 2017b, Winbourne et al., 2017, Dynarski and Houlton, 2018). Higher precipitation could have led to higher nutrient availability e.g. through higher deposition or lateral or vertical transport in the moss carpet and thus, higher nutrient uptake by the mosses.

### 4.2 Nitrogenase activity along moss shoots

We hypothesized that the highest nitrogenase activity would take place in the middle segments of the moss shoots (H2). We based this hypothesis on the assumption that the middle segments would have the optimal conditions in regard to moisture and light for nitrogenase activity. However, our second hypothesis could only be partly confirmed since we found this for only one of the investigated moss species, *P. schreberi*. In contrast, no clear pattern was found for *H. splendens*, even though nitrogenase activity tended to increase with moss shoot depth*.* These differences could be due to different morphologies between the investigated moss species. While *P. schreberi* has a well-defined stem and side branches, *H. splendens* is much more finely branched with several sub-branches that increases water holding capacity along the entire shoot, including the lower parts. This may result in more uniform conditions for nitrogenase activity along *H. splendens* shoots than in *P. schreberi*. Indeed, the moisture content of the moss carpet (5 cm below the moss surface) was found to be higher under *H. splendens* compared to *P. schreberi* at sites near this study (Lett et al., 2024). In addition, nitrogenase activity is generally linked to cyanobacterial colonization (Rousk et al., 2013; Renaudin et al., 2022; Blasi et al., 2025). The lack of clear differences in nitrogenase activity between segments of *H. splendens* therefore might correlate with more uniform cyanobacterial colonization and distribution across the shoot compared to in *P. schreberi*.

Furthermore, even though we collected different moss species from the same habitat, the distinct patterns of nitrogenase activity along the shoots indicated that the distribution of nitrogenase activity is driven more by the moss host and less so by the environment. This could be due to moss species-specific differences in nutrient resorption (Liu et al., 2020a) or other morphological or chemical differences between moss species (Liu and Rousk, 2022). For instance, For instance, *Hylocomium splendens* had higher shoot frequency and higher frequency of leaves per shoot compared to *P. schreberi*, which could promote cyanobacteria colonization and consequently higher nitrogenase activity (Liu and Rousk, 2022).

Our results are in contrast to previous studies that found highest nitrogenase activity in the greenest upper parts of mosses (Smith, 1984, Leppänen et al., 2013). However, these previous studies included what corresponds to all 3 segment depth in our study, into what they more crudely defined as the green upper part of the moss (upper 4 cm) making comparisons difficult. We found a different pattern in nitrogenase activity associated with *H. splendens* than what was reported by Gavazov et al. (2010). Here, they found the highest nitrogenase activity in *H. splendens* in the middle part of the moss, which would correspond to our findings for *P. schreberi*. Yet, *H. splendens* in the previous study was collected from a fen habitat that has very different hydrology compared to a birch forest where our samples originated from, likely also affecting moss shoot morphology. *Hylocomium splenden*s growing in a birch forest will dry out more often, which may prevent cyanobacteria to colonize the top parts of the moss in contrast to a water saturated moss carpet found in a fen.

### 4.3 Nutrient content along moss shoots and correlation with nitrogenase activity

Finally, we wanted to investigate if the nutrients, Fe, P, N and Mo, as well as pH, could explain variation in nitrogenase activity along the shoots. We expected that Fe, P and Mo were positively linked to nitrogenase activity while N and low pH were negatively related to nitrogenase activity (H3). A positive link between nutrients conducive for nitrogenase activity could reflect the demand of the process for these nutrients. Even though we measured total nutrient content, which may not necessarily reflect availability, it can indicate a history of high demand that is reflected in higher nitrogenase activity. Indeed, almost all measured factors, except P, varied between sites with the highest Fe and Mo contents and highest pH in *H. splendens* from the wettest site, which also showed the highest nitrogenase activity. Phosphorus varied little between sites but instead varied greatly between segments and species within sites. pH of both moss species was slightly acidic, but varied little along the moss shoots, which could explain the lack of a link with nitrogenase activity.

The differences we found in nutrient content were significant, but small compared to the wide differences in nitrogenase activity. Given that we found the highest nitrogenase activity in *H. splendens* at the wettest site in segments with the highest content of Mo and Fe, we can partly confirm our third hypothesis (H3). In addition, we found that P was negatively linked to nitrogenase activity at some sites and no correlation between pH and nitrogenase activity, which was inconsistent to our expectation. Yet, the patterns were not consistent across sites, segments and mosses.

The positive relation between Mo and nitrogenase activity is not surprising as it has been shown that Mo availability can influence the nitrogenase activity in mosses from sites close to our sampling sites (Rousk et al., 2017b). On the other hand, the more common form of the nitrogenase enzyme (Mo-based) may not be the prevalent nitrogenase form in all systems as it depends on the availability of nutrients which enzyme form is expressed (e.g. Bellenger et al., 2011). Indeed, indication of the dominance of the complementary V-based nitrogenase has been found in *H. splendens* (Rousk et al., 2017b) and a lack of long-term promotion by Mo additions of nitrogenase activity in the sites close by (Rousk and Rousk, 2020). Further, Mo content of the mosses in our study was less than the proposed threshold of 250 ng Mo g^−1^ (Darnajoux et al., 2019) below which the nitrogenase enzyme shifts from the Mo-based to the vanadium(V)-based isoform. Hence, the lack of a strong link between Mo and nitrogenase activity is not surprising if the alternative nitrogenase prevails in our system.

The negative relation between P and nitrogenase activity was only found in *H. splendens.* These findings are inconsistent with pervious findings that P fertilization had no effects on nitrogenase activity in *H. splendens* (Zackrisson et al., 2009) and a positive effect in *P. schreberi* (Rousk et al., 2017b). This discrepancy suggested that the relation between P and nitrogenase activity can vary between different scales. At a larger scale, increased P availability may enhance overall nitrogenase activity (such as across the entire moss shoot), but this effect can be overridden by other environmental factors such as light availability and the availability of other nutrients like Mo (Rousk et al., 2017b). However, at a smaller scale, a negative relation between P and nitrogenase activity may still occur as high nitrogenase activity has a high requirement for P in the form of ATP. Indeed, *H. splendens* has a limited capacity to absorb new P from the environment (Liu et al., 2020b).

We provided all segments in the study with equal moisture, light and temperature, and hypothesized that nitrogenase activity at different depths along the moss shoot will converge over time if exposed to similar conditions (H4). Under natural conditions, these three factors (moisture, light and temperature) would vary along the moss shoots, making it more likely to find this optimal compromise somewhere along the moss shoots. Surprisingly, contrary to our hypothesis, nitrogenase activity did not converge over time across the segments, as the pattern of different nitrogenase activities along moss shoots held across several weeks.

## 5 Conclusion

Nitrogenase activity in two common feather mosses collected at sites with drastically different precipitation regimes showed clear species-specific differences. There was a strong relationship between nitrogenase activity (i.e., higher capacity for N_2_ fixation) and sites with higher precipitation for *H. splendens,* but not for *P. schreberi*. Nitrogenase activity associated with *P. schreberi* had a clear and consistent pattern along the segments with highest activity in the middle segments 1–2 cm from the moss surface while activity associated with *H. splendens* was overall much higher and showed a higher activity in the bottom segments. Nitrogenase activity varied with measured variables: Fe, P and Mo, less so with N and not with pH. However, variation in nitrogenase activity was far greater and more consistent along the moss shoots, indicating that nutrient content alone cannot explain differences in nitrogenase activity across segments. We showed that nitrogenase activity in mosses can vary significantly at small (moss depth or age) and large scales (different forest sites), and that variation in precipitation can drive orders of magnitude differences in nitrogenase activity in mosses.

Many current efforts to upscale nitrogenate activity to ecosystem N inputs focus on broad environmental drivers such as precipitation and temperature. Our findings showed that nitrogenase activity (thereby the capacity of N_2_ fixation) also varied significantly between moss species and along segments within individual moss shoots. This within-shoot variation may be influenced by microhabitat factors such as nutrient availability, which differ from broader environmental variables. Taken together, the variation of nitrogenase activity and its drivers at small scales should be accounted for in upscaling endeavours, which will improve the accuracy of ecosystem-level N input estimates. Moreover, our findings are also relevant for upscaling approaches that account for seasonal or short-term dynamics, which may influence microhabitat conditions for moss-cyanobacteria association more strongly than the broad climate factors.

## CRediT authorship contribution statement

**Jørgen Ulrik Graudal Levinsen:** Writing – original draft, Visualization, Validation, Methodology, Investigation, Formal analysis, Data curation, Conceptualization. **Mingyue Yuan:** Writing – review & editing, Visualization, Validation, Methodology, Investigation, Formal analysis, Data curation. **Anders Michelsen:** Writing – review & editing, Writing – original draft, Validation, Supervision, Methodology, Investigation, Conceptualization. **Kathrin Rousk:** Writing – review & editing, Writing – original draft, Validation, Supervision, Project administration, Methodology, Investigation, Funding acquisition, Conceptualization.

## Consent to participate

not applicable

## Consent for publication

not applicable

## Ethics approval

Not applicable

## Funding

Funding was provided by the Independent Research Fund Denmark (IRFD) “Research Project 1” (Grant ID: DFF6108–00089B and 4283–00044A), the IRFD “Sapere Aude Starting Grant” (Grant id: 7027–00011B), and the 10.13039/501100000781European Research Council (ERC; grant no. 947719).

## Code availability

The coding used during the study are available from the corresponding author on reasonable request

## Declaration of Competing Interest

The authors declare that they have no known competing financial interests or personal relationships that could have appeared to influence the work reported in this paper.

## Data Availability

The datasets used and/or analysed during the study are available from the corresponding author on reasonable request
