# Supplementary material for: Is moss-associated nitrogen fixation controlled by the same factors across shoots, species and sites?
Source: Environ Exp Bot. 2025 Nov;239:106262. doi: 10.1016/j.envexpbot.2025.106262 (PMC12614486; doi:10.1016/j.envexpbot.2025.106262)
Supplement: Supplementary file 1 — Supplementary material [file mmc1.docx]

**Supporting Table** Summary of fixed effects from the linear mixed-effects model testing the potential difference in on nitrogenase activity (ARA) between weeks. The model included ‘week’ as the fixed effect and sample identity (including species, location, segment) as a random intercept.

| **Effect** | **estimate** | **std.error** | **statistic** | **df** | **p.value** |
| --- | --- | --- | --- | --- | --- |
| (Intercept) | 7.840 | 7.074 | 1.108 | 1 | 0.478 |
| week1 | 0.000 | 0.530 | 0.000 | 520 | 1.000 |
| week2 | 0.000 | 0.530 | 0.000 | 520 | 1.000 |
| week3 | 0.000 | 0.530 | 0.000 | 520 | 1.000 |
| week4 | 0.000 | 0.530 | 0.000 | 520 | 1.000 |
| week5 | 0.000 | 0.530 | 0.000 | 520 | 1.000 |

**Supporting figure 1** Relationship between nitrogenase activity (ethylene production. nmol g dw^-1^ hr^-1^) and concentrations of nitrogen (N). phosphorus (P). iron (Fe) and molybdenum (Mo) (n=6) in three different moss segments (“Top”. “Middle” and “Bottom”) from two different species of feather moss . *Pleurozium schreberi* and *Hylocomium splendens.*  These two mosses were collected along steep precipitation gradient in Subarctic birch forests (Abisko “dry”. Låktatjåkka “med” and Katterjåkk “wet”). Regressions with solid lines included are significant at P < 0.05. and those with dashed lines are significant at P < 0.10)


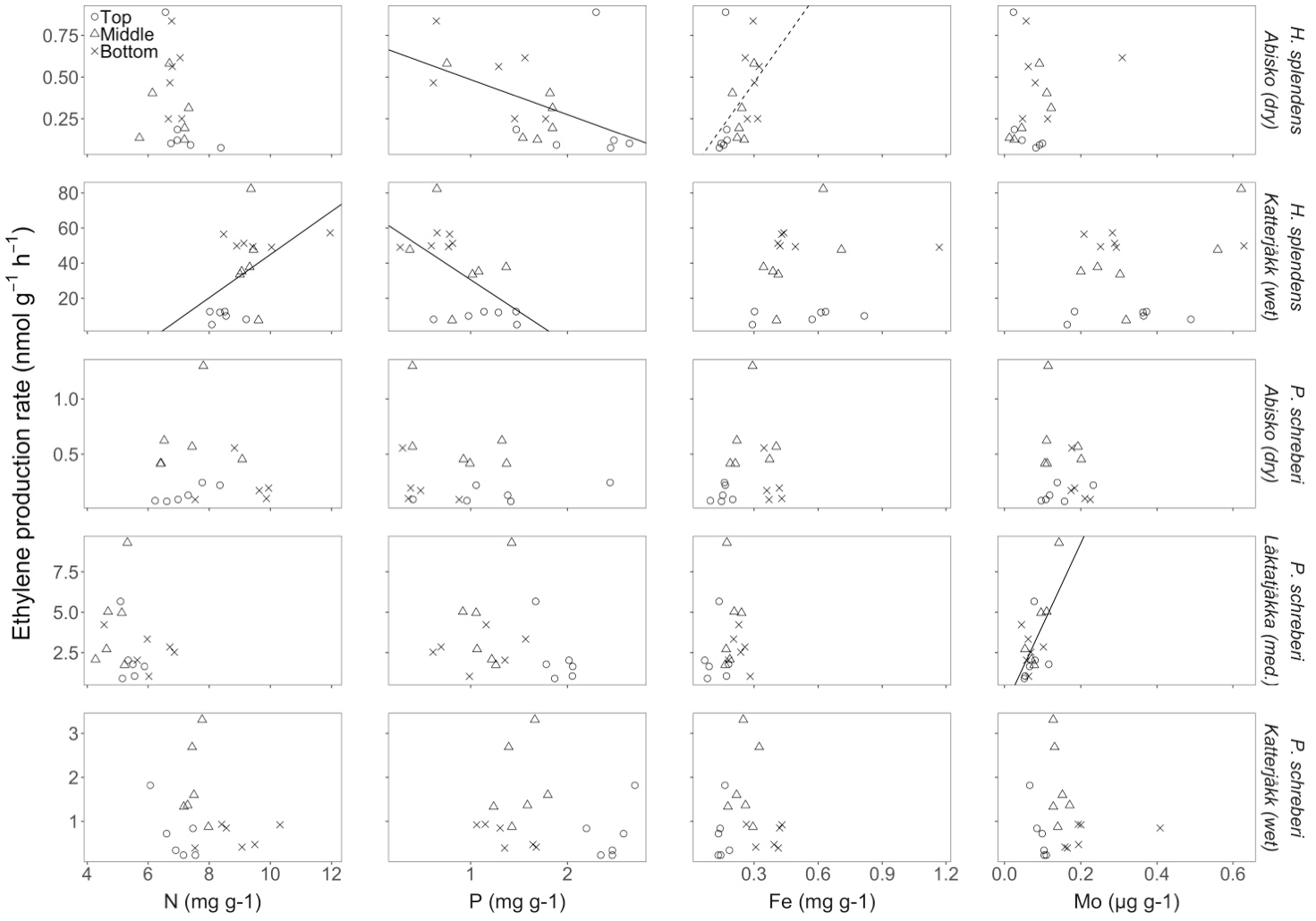


**Supporting figure 2** Relationship between nitrogenase activity (ethylene production. nmol g dw^-1^ hr^-1^) and mean annual precipitation in three moss segments (“Top”. “Middle” and “Bottom”) from two different species of feather moss. *Pleurozium schreberi* and *Hylocomium splendens*.

**
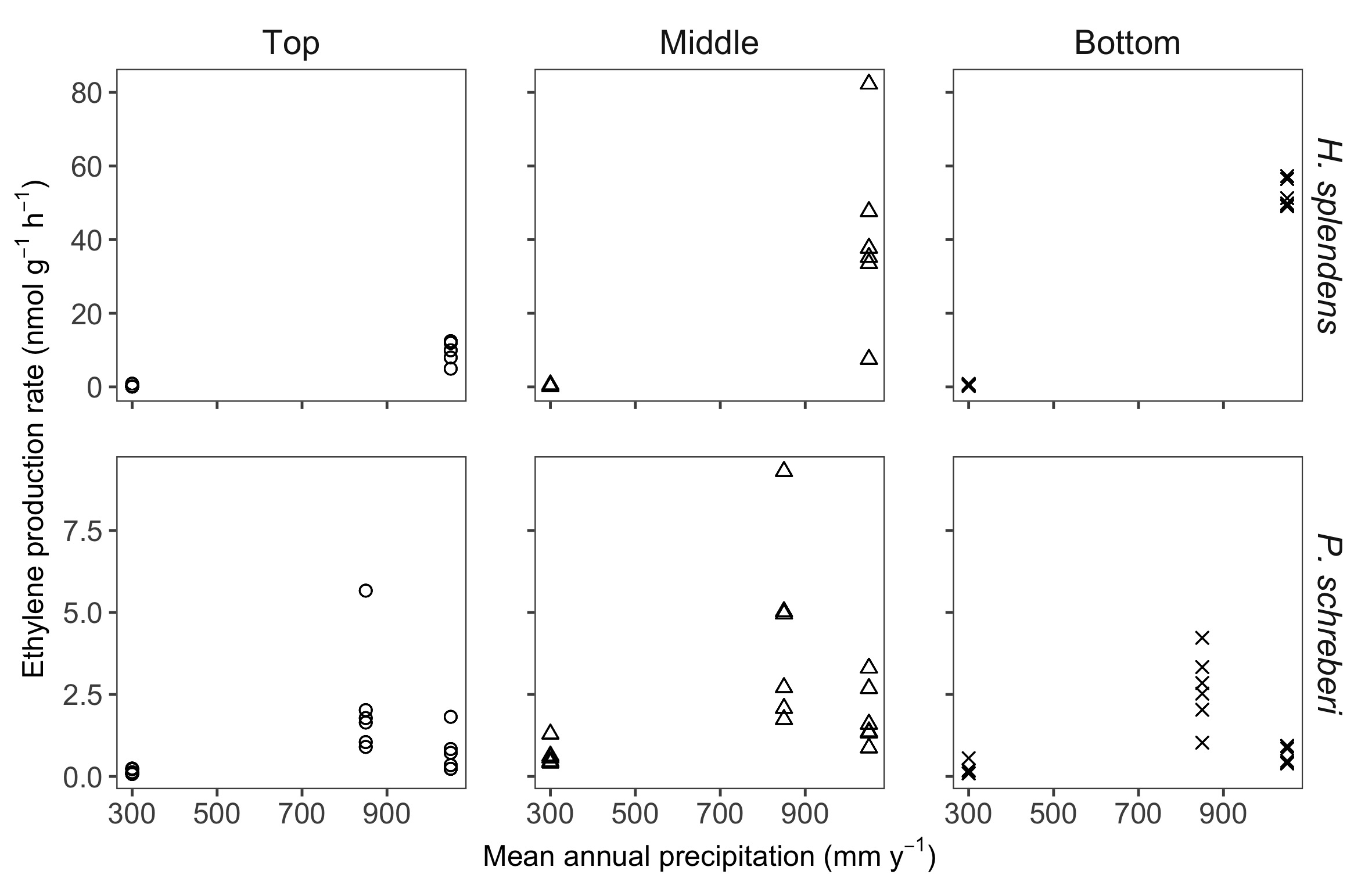
**
